# A Combined Factor V and Factor VIII Deficiency: A Case Report

**DOI:** 10.7759/cureus.26568

**Published:** 2022-07-05

**Authors:** Ammar Kalas, Abdelrahman Hassan, Oumar Alkhalifa, Majd Dali, Fawaz Shaheen

**Affiliations:** 1 College of Medicine, Sulaiman Al Rajhi University, Al Bukayriyah, SAU; 2 Internal Medicine, King Saud Hospital, Unayzah, SAU

**Keywords:** bleeding disorder, combined, clotting factor v deficiency, clotting factor viii deficiency, coagulopathy

## Abstract

Factor V and VIII deficiency (F5F8D) is a rare coagulopathy; it’s an autosomal recessive condition. This case report is about of 15-year-old unmarried Saudi female, who presented with a complaint of pain in the lower abdomen. Complete blood count and coagulation profile revealed low hemoglobin and prolongation of activated partial thromboplastin time (aPTT), prothrombin time (PT), and international normalized ratio (INR). The deficiency of factor V and factor VIII was confirmed with factor test revealing reduced activities of factor V and VIII Combined deficiency of factors V and VIII should be considered in differential diagnoses of patients with prolonged INR, PT, and aPTT. Medical management is reserved for those who present with significant bleeding.

## Introduction

The combined factor V and factor VIII deficiency (F5F8D) is an autosomal recessive constitutional hematological disorder that is relatively uncommon, the condition was first identified in 1954 by Oeri et al. [1]. The estimated prevalence ranges from 1 in 100,000 in Middle Eastern countries compared to one in 1 million in other regions of the world. A considerable number of these cases belong to the Mediterranean and Asia areas, particularly the countries of the Middle East. The affected genes must be carried by patients’ parents in a heterozygous genotype pattern. Thus, the incidence in regions that have higher rates of consanguineous marriages seems to be more prevalent. The presentation usually is a bleeding tendency that varies in severity between mild to moderate. Easy bruising, epistaxis, menorrhagia, bleeding after dental surgery, gum bleeding, and soft-tissue bleeding are some of the symptoms [2].

Bleeding time and platelet count appear normal while international normalized ratio (INR) and activated partial thromboplastin time (aPTT) are observably prolonged. Mixing studies show the correction of prothrombin time (PT) and aPTT. To reach a diagnosis, further factor testing should be performed. Advanced genetic analysis could be done to identify the exact gene mutation responsible for the case. The successful management to prevent bleeding from surgery includes the administration of 1-deamino-8-d-arginine vasopressin (DDAVP and fresh frozen plasma [3].

## Case presentation

A 15-year-old Saudi unmarried female presented to the emergency department with periumbilical pain, imaging studies demonstrated a ruptured ovarian cyst with mild-to-moderate fluid within the pelvis. During preoperative workup for a laparotomy owing to the aforementioned diagnosis, asymptomatic prolongation of PT, INR, and aPTT was reported. Medical history in the past was significant for bleeding from the gums and profuse bleeding following the dental extraction procedure. She had no history of spontaneous bleeding, muscle or joint bleeding. A routine physical examination revealed nothing unusual. Additionally, her mother and father are relatives. Laboratory investigations (Table 1) were performed. Hematological investigations showed normal platelet count and normal peripheral smear except for microcytic hypochromic anemia, PT of 26.3 seconds, INR of 2.09, and aPTT of 105.6 seconds. The mixing study resulted in the correction of PT, INR, and aPPT.

**Table 1:** Laboratory investigation Hb: hemoglobin; WBC: white blood count; APTT: activated partial thromboplastin time; PT: prothrombin time INR: international normalized ratio

Laboratory investigation	Patient value	Reference value
Hb	10.6 g/dL	11.9-15
WBC	8.1 × 10^9^/L	4-10
Platelet	263 × 10^9^/L	150-400
aPTT	105.6 seconds	20.4-33.87
PT	26.3 seconds	10.60-14.19
INR	2.09 seconds	0.89-1.16
Iron	4 µmol/L	11-29
Alkaline phosphates	116 U/L	44-147
Na	144 mmol/L	135-145
K	3.7 mmol/L	3.5-5
Glucose (random)	7.37 mmol/L	4.4-7.8
Bilirubin (conjugated)	1.7 µmol/L	<5.1
Creatinine	59 µmol/L	53-97.2
Uric acid	377 µmol/L	130-378
Mixing study	Corrected PT, aPTT, INR	-
Indirect Coombs test	Negative	-
Direct Coombs test	Negative	-

The patient has received prophylaxis with fresh frozen plasma and packed red blood cells (PRBC) prior to, during, and after surgery due to persistently elevated PT, aPTT, and INR. Moreover, vitamin K and tranexamic acid were given. Her postoperative course was unremarkable with no bleeding. She was referred to a higher care center to do factor assays to establish a diagnosis. So, she conducted the factors assay in King Faisal Specialist Hospital & Research Center in Riyadh, and the results (Table 2) showed reduced activity of factors V and VIII. A combination of factor V and factor VIII deficiency was established, and the patient was asked to come in on a regular visit. It was also recommended in case of bleeding and/or need for surgery, factor VIII and two units of fresh frozen plasma (FFP) to be given as needed.

**Table 2:** Factor assays

Coagulation test	Absolute values	Reference value
Factor II activity	82%	80-120
Factor V activity	8%	80-120
Factor VII activity	69%	65-150
Factor VIII activity	6%	80-120
Factor IX activity	74%	70-140
Factor X activity	74%	80-120
Factor XII activity	77%	80-120
Anti-thrombin III	107%	80-120
Fibrinogen level	6.46 g/ L	1.4-4.4
D-dimer	1.78 µg/mL FEU	0-0.5
Protein C functional	0.92 IU/mL	0.5-1.24
Protein S functional	0.75 IU/mL	0.6-1.40

## Discussion

Congenital combined factor V and factor VIII deficiency is a form of peculiar autosomal recessive bleeding disorder which is more common in places where kindred marriages are more common. Combined factor V and factor VIII deficiency is the most common kind of combined plasma coagulation factor deficits. Families impacted have been reported from all over the world, including the Middle East, North America, Europe, and the Far East. However, Middle Eastern heritage has the highest prevalence of this illness [2].

The disorder results from gene mutations in either two genes LMAN1 (chromosome 18; 18q21) or MCFD2 (chromosome 2; 2p21). The two genes produce the ERGIC-53/MCFD2 protein complex, which serves as a cargo receptor, allowing coagulation factors V and VIII to be transported to the Golgi apparatus, coming from the endoplasmic reticulum. LMAN1 mutations explain around 70% of cases and solely comprise null mutations. MCFD2 mutations occur in about 30% of instances and include both null and missense variants [3,4]. Unfortunately, due to lack of adequate, genetic screening for both these mutations was not done for our patient.

A screening correlogram and factor assay are used to diagnose this disease. Factor V and factor VIII levels should be between 60% and 150% in the normal range. However, in F5F8D levels usually range from 1% to 46%, but most patients have levels between 5% and 30%. The levels of factor V and factor VIII in our patients is 8% and 6%, respectively. Diagnosis is usually considered after the discovery of prolonged activated partial thromboplastin and prothrombin times [2,3]. We reported this case since, according to our literature study, the Middle East is the most frequent site for this condition. As a result, almost any clinician may encounter individuals who suffer from this condition.

The simultaneous reduction in plasma levels of factor V and factor VIII causes mild-to-moderate manifestations. Symptoms of bleeding vary; however, they are often comparable to those of single factor V or VIII insufficiency. Epistaxis, menorrhagia, easy bruising, bleeding following trauma or surgery, and to a lesser extent, hemarthrosis and muscle hematomas are the most prevalent symptoms [4,5]. Gum bleeds and prolonged bleeding following a tooth extraction have all occurred previously for our patient. However, there was no evidence or signs of menorrhagia, epistaxis, or gastrointestinal bleeding.

Regular prophylaxis is not necessary for combination F5F8D, and patients are typically not treated until therapy is required; nevertheless, prophylaxis should indeed be considered in situations of recurrent hemarthrosis or intramuscular hemorrhage. Replacement of the factors is the therapy. FFP as a therapy regimen that can give factor V and factor VIII concentrate, has been suggested by the Hemophilia Centre Doctors’ Organization in the United Kingdom. Particular factor concentrates, whether plasma-derived or recombinant, are superior in replacing factor VIII. Published investigations have shown that an FFP-containing regimen can effectively limit bleeding during tooth extractions, circumcision, and labor preparation. Desmopressin can still be utilized to treat minor bleeds under certain circumstances [5,6].

The overall prognosis for mild instances of the illness is good. Patients with more severe varieties of the disease shall be properly treated in a highly specialized hospital [3].

## Conclusions

The combined deficiency of factor V and factor VIII is more prevalent in areas where consanguinity is common such as middle eastern countries and the Mediterranean region. The disorder should be suspected in any patient who presents with prolonged PT and aPTT and meets the epidemiologic features. Management in form of regular replacement with FFP and factors concentrate is reserved for recurrent severe bleeding.
